# Novel Bifunctional Acylase from *Actinoplanes utahensis*: A Versatile Enzyme to Synthesize Antimicrobial Compounds and Use in Quorum Quenching Processes

**DOI:** 10.3390/antibiotics10080922

**Published:** 2021-07-29

**Authors:** Lara Serrano-Aguirre, Rodrigo Velasco-Bucheli, Begoña García-Álvarez, Ana Saborido, Miguel Arroyo, Isabel de la Mata

**Affiliations:** Department of Biochemistry and Molecular Biology, Faculty of Biology, Universidad Complutense de Madrid (UCM), José Antonio Nováis 12, E-28040 Madrid, Spain; larase01@ucm.es (L.S.-A.); rvelasc@unal.edu.co (R.V.-B.); begoga01@ucm.es (B.G.-Á.); asaborid@ucm.es (A.S.); marroyos@ucm.es (M.A.)

**Keywords:** *N*-acyl-homoserine lactone, *N*-acyl-homoserine lactone acylase, penicillin acylase, Ntn-hydrolase, quorum sensing, quorum quenching, *Actinoplanes utahensis*

## Abstract

Many intercellular communication processes, known as quorum sensing (QS), are regulated by the autoinducers *N*-acyl-l-homoserine lactones (AHLs) in Gram-negative bacteria. The inactivation of these QS processes using different quorum quenching (QQ) strategies, such as enzymatic degradation of the autoinducers or the receptor blocking with non-active analogs, could be the basis for the development of new antimicrobials. This study details the heterologous expression, purification, and characterization of a novel *N*-acylhomoserine lactone acylase from *Actinoplanes utahensis* NRRL 12052 (*Au*AHLA), which can hydrolyze different natural penicillins and *N*-acyl-homoserine lactones (with or without 3-oxo substitution), as well as synthesize them. Kinetic parameters for the hydrolysis of a broad range of substrates have shown that *Au*AHLA prefers penicillin V, followed by C_12_-HSL. In addition, *Au*AHLA inhibits the production of violacein by *Chromobacterium violaceum* CV026, confirming its potential use as a QQ agent. Noteworthy, *Au*AHLA is also able to efficiently synthesize penicillin V, besides natural AHLs and phenoxyacetyl-homoserine lactone (POHL), a non-natural analog of AHLs that could be used to block QS receptors and inhibit signal of autoinducers, being the first reported AHL acylase capable of synthesizing AHLs.

## 1. Introduction

*N*-acyl-l-homoserine lactones (AHLs) are small signal molecules involved in intercellular communication processes, known as quorum sensing (QS), in Gram-negative bacteria. QS allows a bacterial community to synchronize the expression of a set of target genes in order to respond to environmental changes in a density-dependent manner [1]. Several biological functions, such as secretion of virulence factors and biofilm formation, are regulated by this mechanism in many pathogenic microorganisms [2,3,4]. AHLs are composed of the homoserine lactone ring (HSL) and an acyl chain with a different number of carbons, saturation, or side-chain substitution [5]. Quorum quenching (QQ) processes consist of the interference of QS by inhibition of the biosynthesis or detection of signal molecules or by enzymatic modification or degradation of the autoinducers [6]. Among QQ enzymes, the main groups of AHL-degrading or modifying enzymes are lactonases, which catalyze the hydrolysis of the lactone ring; acylases, which catalyze the hydrolysis of the amide bond to form the homoserine lactone ring (HSL) and the corresponding fatty acid; and oxidoreductases, which reduce the carbonyl to hydroxyl [7,8,9]. QQ enzymes could be used for biotechnological applications such as antivirulence tool in the control and treatment of bacterial infections [10] and decreasing of biofouling in membrane bioreactors [11,12,13]. Another QQ approach consists in AHLs analogs that form non-active signal-receptor complexes. Multiple studies have investigated this strategy, including modified AHLs, non-natural AHLs and non-AHL scaffold compounds [6,14].

Fatty acids and HSL released by AHL acylases cannot form a functional QS signal spontaneously, and they can be used as carbon and nitrogen source [15]. Most of the described and characterized AHL acylases belong to the N-terminal nucleophile (Ntn) hydrolase superfamily with an αββα fold and a nucleophilic catalytic residue in the N-terminal [16]. These enzymes are synthesized as a single polypeptide chain with a signal peptide, an α-subunit, a spacer peptide, and a β-subunit [17]. The α- and β-subunits are about 20 and 60 kDa, respectively [18]. Other characteristic enzymes of this superfamily are β-lactam acylases such as penicillin acylases, having reported AHL acylase activity for some of them [19,20]. Penicillin acylases are commonly used for the synthesis of β-lactam antibiotics such as semisynthetic penicillins, by deacylation of natural penicillins and acylation of 6-aminopenicillanic acid (6-APA) using non-natural side chains [21,22]. However, none acylase able to synthesize AHLs has yet been described.

In the present study, we report the heterologous expression and characterization of a novel bifunctional acylase from *Actinoplanes utahensis* NRRL 12052 (*Au*AHLA) and its hydrolytic activity against different natural penicillins and *N*-acyl-homoserine lactones, evaluating its possible role as a QQ agent. More interesting, we demonstrate its capacity to carry out the enzymatic synthesis of penicillin V, as well as natural AHLs and non-natural analogs of AHLs that could be used in QQ processes, being the first example of AHLs enzymatic synthesis catalyzed by an acylase.

## 2. Results

### 2.1. Identification of the Putative ahla Gene in the Genome of Actinoplanes utahensis NRRL 12052

**Figure 1:**
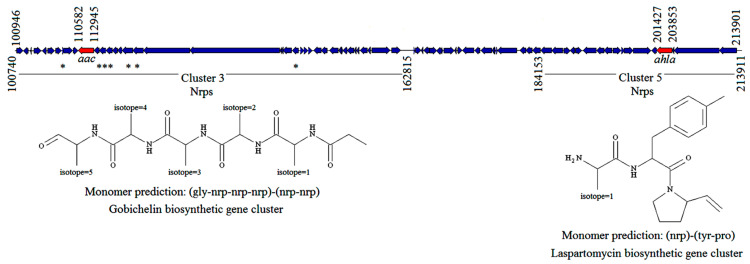
Clusters 3 and 5 within the contig 8-containing *aac* and *ahla* genes (highlighted in red), and rough prediction of core scaffolds produced by those clusters based on assumed PKS/NRPS collinearity without considering tailoring reactions. Genes highlighted with asterisks are related to ABC-transporter systems. The predicted gene clusters contain the domain annotations and the prediction of putative core structures. In both cases, the role of these acylases seems to be relevant in the synthesis of nonribosomal peptides.

The presence of the putative *ahla* gene was detected in the draft genome of *Actinoplanes utahensis* NRRL 12052 [23]. This gene, as well as the *aac* gene, which encodes for the aculeacin A acylase from *A. utahensis* (*Au*AAC) [24], was located within contig 8 and related to nonribosomal peptide synthetases (NRPS) and siderophores biosynthesis based on antiSMASH analysis [25]. In particular, *ahla* gene was identified within cluster 3 and was related to gobichelin biosynthesis, while *aac* gene was identified within cluster 5 and was related to laspartomycin biosynthesis (Figure 1).

The amino acid sequence of this putative *Au*AHLA deduced from the *ahla* gene has 808 residues, with a predicted signal peptide of 33 amino acids, according to SignalP predictor [26] and α- and β-subunits of 21.6 and 59.8 kDa, respectively, according to ExPASy ProtParam tool [27]. *Au*AHLA keeps 44% of identity with *Au*AAC [24,28], 51% with penicillin V acylase from *Streptomyces lavendulae* (*Sl*PVA) [29], 48% with penicillin V acylase from *Streptomyces mobaraensis* (*Sm*PVA) [30], 50% with acylhomoserine lactone acylase from *Streptomyces* sp. M664 (AhlM) [31], and 50% with cyclic lipopeptide acylase from *Streptomyces* FERM-BP5809 (*Ss*CLA) [32] (Supplementary Material, Figure S1). Moreover, sequence alignment analysis by COBALT [33] using *Au*AHLA, *Au*AAC, *Sl*PVA, and other 174 acylases indicated the presence of conserved essential amino acids involved in catalysis and substrate binding (Supplementary Material, Table S1).

### 2.2. Heterologous Expression and Purification of AuAHLA

**Figure 2:**
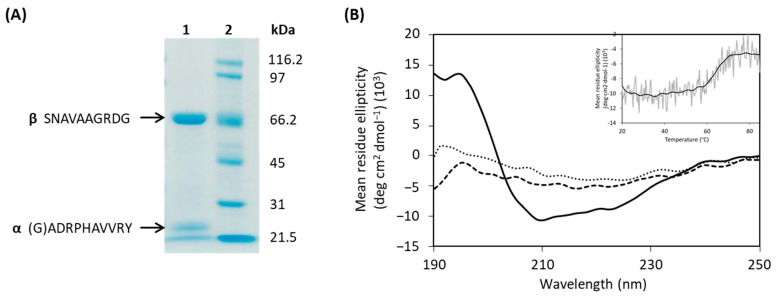
(**A**) SDS-PAGE analysis of purified *Au*AHLA produced by the recombinant strain *Rhodococcus* pENV19*ahla*. Lane 1, purified *Au*AHLA; lane 2, standard molecular mass markers. (**B**) Far-UV CD spectra of *Au*AHLA 0.07 mg/mL at 20 °C (solid line), 85 °C (dashed line) and 20 °C after thermal unfolding (dotted line). Inset shows thermal unfolding of *Au*AHLA at 208 nm between 20 and 85 °C.

The putative *ahla* gene was amplified by PCR, cloned in the pENV19, and the recombinant strain *Rhodococcus* pENV19*ahla* was obtained as described in the Materials and Methods section. After 72 h at 30 °C, penicillin acylase activity was detected in the recombinant strain culture supernatants, indicating that the signal peptide of native *Au*AHLA was correctly recognized by *Rhodococcus*. Recombinant *Au*AHLA was purified 19-fold from the cell-free broth by only one chromatographic step followed by an ultrafiltration step (Supplementary Material, Table S2), being a considerably fast procedure. The SDS-PAGE analysis of purified *Au*AHLA (Figure 2A) showed that the enzyme is a heterodimer composed of two subunits, and the molecular mass determined by MALDI-TOF for α- and β-subunits were 20 and 66.4 kDa, respectively (Supplementary Material, Figure S2A). Moreover, the identity of α- and β-subunits detected in the SDS-PAGE was confirmed by peptide mass fingerprinting of each band (Supplementary Material, Figure S2B). The N-terminal amino acid sequences determined for α- and β-subunits were (G)ADRPHAVVR and SNAVAAGRDG, respectively. These results confirmed that *Rhodococcus* produces the mature enzyme extracellularly, as well as the presence of a signal peptide of 34–35 amino acids.

### 2.3. Characterization of Recombinant AuAHLA

After its purification, the recombinant *Au*AHLA was structurally and functionally characterized. First, its molar extinction coefficient was determined by the method of Edelhoch [34], being ε^280^ = 111,302 M^−1^ cm^−1^. The homogeneity and oligomeric state of *Au*AHLA was studied by analytical ultracentrifugation (Supplementary Material, Figure S2D). Sedimentation coefficients were determined from the analysis of sedimentation velocity experiments, and the presence of the main species with an *S* value of 4.8 was observed. This value is compatible with the behavior of a globular protein with a molecular weight of 66.2 kDa, being a smaller size than the molecular weight determined by MALDI-TOF analysis for *Au*AHLA. This result revealed that this protein probably shows a spheroid oblate-shape with different hydrodynamic properties [35], as previously observed for *Sl*PVA and *Au*AAC (unpublished data).

**Figure 3:**
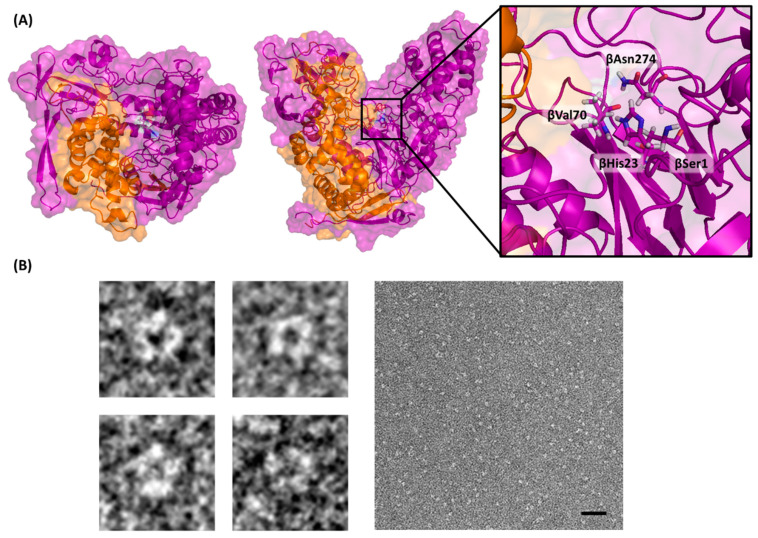
Tridimensional structure analysis of *Au*AHLA. (**A**) Predicted 3D heterodimeric structure model of *Au*AHLA, showing α-subunit in orange and β-subunit in purple. Top view, side view, and putative active-site catalytic amino acids (βSer1, βHis23, βVal70, and βAsn274) are shown. (**B**) Electron microscopy micrographs of negatively stained *Au*AHLA. A nominal magnification of 50,000, scale bar 50 nm. Left, representative *Au*AHLA particles with an approximate diameter of 8 nm, and right, image of monodisperse and uniform *Au*AHLA sample.

The secondary structure of *Au*AHLA was analyzed by CD spectroscopy in the far-UV region (Figure 2B). *Au*AHLA contains 31% α-helix, 18.7% β-sheet, 17.4% β-turn, and 33.7% random coil structure. Moreover, CD thermal denaturation revealed that *Au*AHLA was thermostable, showing a melting temperature (T_m_) of 64 °C (Figure 2B, inset). The predicted 3D structure bioinformatic model of *Au*AHLA and its putative catalytic amino acids (βSer1, βHis23, βVal70, and βAsn274) are shown in Figure 3A. In addition, the shape of purified *Au*AHLA was analyzed by negative-stain electron microscopy (Figure 3B), revealing the presence of monodisperse and uniform molecules with a diameter of about 8 nm, consistent with the size of *Au*AHLA heterodimers, which probably correspond to a top view of the enzyme (Figure 3A, left). These particles present a hole in the middle of them, where catalytic amino acids are presumably located, and substrate could be fitted.

**Figure 4:**
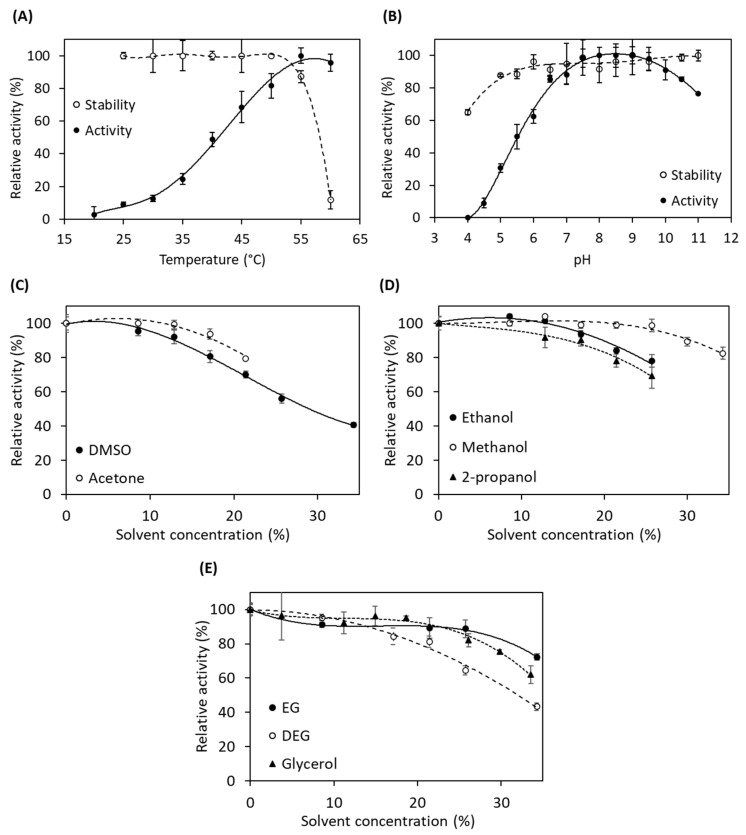
Biochemical characterization of *Au*AHLA. Effect of temperature (**A**) and pH (**B**) on *Au*AHLA activity and stability. Effect of organic co-solvents on *Au*AHLA activity: aprotic polar solvents (**C**), alcohols (**D**), and glycerol and glycols (**E**).

Furthermore, the effect of temperature, pH, and organic co-solvents on the acylase activity of *Au*AHLA was studied. The enzyme was quite stable at temperatures below 50 °C and at a wide pH range, between 6 and 11, and it showed maximum activity at 55 °C and pH values between 7.5 and 9.5 (Figure 4A,B). Therefore, the optimum reaction temperature and pH for further experiments were set at 45 °C and pH 8.0. The activation energy was also calculated using the Arrhenius equation, being 53.3 kJ mol^−1^. Moreover, the activity of *Au*AHLA was not affected by the presence of NaCl at concentrations below 1 M (data not shown).

Acylase activity of *Au*AHLA was also assayed in the presence of different concentrations of organic co-solvents (Figure 4C–E). The addition of aprotic polar solvents resulted in a decrease in acylase activity at around 80% at 15% (*v*/*v*) DMSO and 20% (*v*/*v*) acetone (Figure 4C), while in the presence of alcohols and polyols, acylase activity was quite stable until 15% (*v*/*v*) ethanol and 2-propanol and 25% (*v*/*v*) methanol (Figure 4D). The enzyme kept its acylase activity until 25% (*v*/*v*) glycerol and ethylene glycol (EG), while its activity decreased at 60% at the same concentration of diethylene glycol (DEG) (Figure 4E).

### 2.4. Kinetic Parameters of AuAHLA for the Hydrolysis of Different Substrates

Substrate specificity of *Au*AHLA was determined by calculating the kinetic parameters for the hydrolysis of several penicillins and enantiopure *N*-acyl-l-homoserine lactones (Table 1) using the spectrofluorimetric assay described in the Materials and Methods section. *Au*AHLA was able to hydrolyze different penicillins, being penicillin V the best substrate, followed by penicillin dihydroF. In the case of aliphatic AHLs, *Au*AHLA showed the highest catalytic efficiency with C_12_-HSL, followed by C_10_-HSL. When 3-oxo-AHLs were used as substrate, the catalytic efficiency is drastically decreased with respect to the homologous AHLs with aliphatic acyl chains. These results suggest that the 3-oxo group has a negative impact on enzyme-substrate interaction. Moreover, kinetic parameters for C_4_-HSL and 3-oxo-C_6_-HSL could not be determined, and for 3-oxo-C_8_-HSL, the catalytic efficiency was poor. Acylase activity of *Au*AHLA was also tested using aculeacin A and caspofungin as substrates, but no hydrolysis was observed (data not shown).

### 2.5. Synthetic Activity of AuAHLA

**Figure 5:**
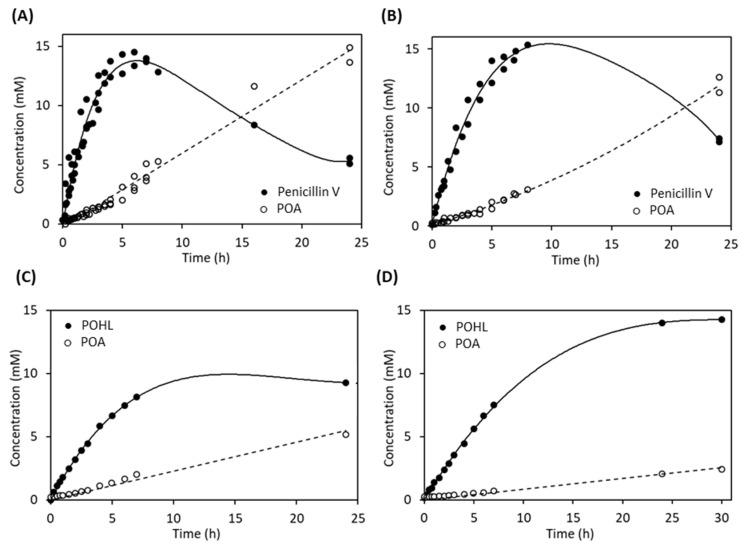
Synthetic activity of *Au*AHLA. Enzymatic synthesis of penicillin V catalyzed by *Au*AHLA in the presence of 3% (**A**) and 32% (*v*/*v*) DMSO (**B**) as co-solvent. Enzymatic synthesis of POHL catalyzed by *Au*AHLA in the presence of 23% (**C**) and 50% (*v*/*v*) DMSO (**D**) as co-solvent. Reaction conditions: 1 IU/mL *Au*AHLA, 0.1 M potassium phosphate pH 7.0 buffer, 100 mM acyl acceptor (6-APA or HSL), 20 mM MPOA and 30 °C.

In addition to the analysis of the hydrolytic activity of *Au*AHLA using different substrates, we evaluated if this enzyme was also able to catalyze the synthesis reaction of those compounds in the appropriate reaction conditions. Kinetically controlled synthesis of penicillin V catalyzed by *Au*AHLA was carried out using 6-APA as acyl acceptor and MPOA as acyl donor. The effect of enzyme concentration and DMSO as co-solvent were studied. As it might be expected, synthetic activity (V_s_) and maximum yield (Y_max_) were increased as enzyme concentration was increased (Table 2) and reaction time for Y_max_ using 1 IU/mL *Au*AHLA was shorter than using 0.125 or 0.25 IU/mL *Au*AHLA. Figure 5A,B show the time-course reaction profiles using 1 IU/mL *Au*AHLA in the presence of 3% and 32% (*v*/*v*) DMSO, respectively. The percentage of DMSO did not affect the synthetic activity (V_s_) of *Au*AHLA (Table 2), whereas the hydrolytic activity (V_h_) was decreased when the concentration of DMSO in the reaction mixture was increased. These results agree with the previously observed reduction in penicillin V acylase activity in the presence of DMSO (Figure 4C). Because of this selective decrease in hydrolytic activity, the S/H ratio was increased, reaching a slightly higher Y_max_ value in a longer reaction time than in the presence of 3% (*v*/*v*) DMSO. These differences could be due to the reduction in water activity (a_w_) of the system in the presence of DMSO as a co-solvent [36].

After observing that *Au*AHLA was able to catalyze the enzymatic synthesis of penicillin V efficiently, its synthetic activity using L-HSL as acyl acceptor was studied. First, MPOA was used as an acyl donor. The synthesized AHLs analog, named phenoxyacetyl-HSL (POHL), was previously described and obtained by chemical procedures, as well as several derivatives with different substitutions [37]. These non-natural AHLs analogs, named aryl-L-homoserine lactones, can be used as antagonists for QS receptors to inhibit QS-regulated processes. Due to the low solubility of AHLs in water, POHL synthesis reactions catalyzed by *Au*AHLA were carried out in the presence of 23% and 50% (*v*/*v*) DMSO (Figure 5C and Figure 5D, respectively), and parameters for each reaction are shown in Table 2. While V_s_ is similar in both conditions, V_h_ is 3-fold lower when DMSO was increased from 23% to 50% (*v*/*v*), leading to a higher S/H ratio and Y_max_. Although V_s_ is lower than for the synthesis of penicillin V, *Au*AHLA can catalyze the enzymatic synthesis of POHL, reaching a similar Y_max_.

Moreover, enzymatic synthesis of natural AHLs was tested, using different fatty acid methyl esters as acyl donors and L-HSL as acyl acceptors. The molecular structure of synthesized AHLs was confirmed by LC-MS/MS analysis (Supplementary Material, Table S3). Due to the disadvantages of the chemical synthesis of these compounds, such as chemical wastes, long synthetic routes, and product purification processes, the enzymatic synthesis could be a very interesting alternative to obtain natural AHLs and reduce costs of AHL-based QS research.

### 2.6. Bioassay Activity of Hydrolyzed and Synthetized AHLs by AuAHLA

**Figure 6:**
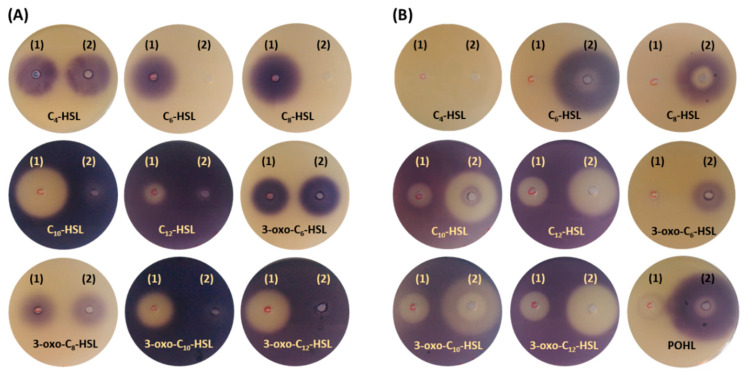
*Chromobacterium violaceum* CV026 bioassays testing the hydrolysis of AHLs (**A**) and the enzymatic synthesized AHLs and POHL by *Au*AHLA (**B**). In each agar plate, spot (1) contains the reaction mixture without *Au*AHLA, and spot (2) contains the enzymatic reaction mixture.

AHL acylase activity of *Au*AHLA was also tested by the bioassay based on violacein production by *Chromobacterium violaceum* CV026 to confirm if this enzyme could be used in quorum quenching processes. The synthesis of violacein by *C. violaceum* CV026 is induced by AHLs with acyl chains from C_4_ to C_8_, while this process is inhibited by AHLs with acyl chains from C_10_ to C_14_. As it is shown in Figure 6A, *Au*AHLA was able to inhibit violacein production using C_6_-HSL and C_8_-HSL because of their efficient enzymatic hydrolysis (forward bioassay). Regarding 3-oxo-C_6_-HSL and 3-oxo-C_8_-HSL, enzymatic hydrolysis was not observed. In contrast to this, violacein production by *C. violaceum* CV026 was induced in all reverse bioassay plates, indicating that *Au*AHLA was able to efficiently hydrolyze AHLs with long acyl chains both aliphatic- and 3-oxo substituted. These results, together with the kinetic parameters determined for the hydrolysis of AHLs (Table 1), demonstrate that *Au*AHLA has AHL acylase activity and suggest its potential use as a QQ agent to disrupt QS processes.

Furthermore, the bioactivity of non-natural POHL and natural AHLs synthesized by *Au*AHLA was also qualitatively analyzed using *C. violaceum* CV026 (Figure 6B). No synthetic activity for C_4_-HSL was observed, whereas enzymatic synthesized POHL, C_6_-HSL, C_8_-HSL, and 3-oxo-C_6_-HSL induced the production of violacein. Likewise, enzymatically synthesized C_10_-HSL, C_12_-HSL, 3-oxo-C_10_-HSL, and 3-oxo-C_12_-HSL inhibited the production of violacein by *C. violaceum* CV026 in the presence of C_6_-HSL as autoinducer.

## 3. Discussion

The *ahla* gene from *Actinoplanes utahensis* NRRL 12052 has been successfully cloned and extracellularly expressed in the recombinant strain *Rhodococcus* pENV19*ahla*, showing that it encodes for the acylase *Au*AHLA, which has been correctly post-translational processed. This fact demonstrates that *Rhodococcus* is an adequate expression host for actinomycetes’ genes [29,38,39]. Moreover, the resulting mature and active enzyme has been purified in a single chromatographic step.

N-terminal amino acid sequence analysis has confirmed that *Au*AHLA belongs to the Ntn-hydrolase superfamily with a heterodimeric structure and a serine as nucleophilic catalytic residue. Moreover, sequence alignment analysis has shown that putative catalytic amino acids are highly conserved with respect to other acylases (Supplementary Material, Figure S1 and Table S1).

Based on phylogenetic analysis, *Au*AHLA belongs to the AHL acylase group A within the Ntn-hydrolase superfamily (Supplementary Material, Figure S3), such as other related acylases: *Au*AAC from *A. utahensis* [24,28], *Sm*PVA from *Streptomyces mobaraensis* [30], *Sl*PVA from *S. lavendulae* [29,40], *Ss*CLA from *Streptomyces* sp. FERM-BP5809 [32], and AhlM from *Streptomyces* sp. M664 [31]. Other members of this group are: QqaR from *Deinococcus radiodurans* [41], Aac from *Ralstonia solanacearum* [42], AiiD from *Ralstonia* sp. XJ12B [43], AhaP from *Psychrobacter* sp. M9-54-1 [44], MacQ from *Acidovorax* sp. MR-S7 [45], HacA from *Pseudomonas syringae* B728a [46], PvdQ from *P. aeruginosa* PAO1 [47], and Aac from *Shewanella* sp. [48]. This AHL acylase group A is distant from the AHL acylase group B, which is closer to the PGA group. A separate cluster is formed by the cholylglycine hydrolase (CGH) group, together with the amidase group. CGH group includes PVAs and bile salt hydrolases (BSHs), having significant sequence, structural and catalytic mechanism similarities [49]. Though, the acylases Slac1 and Slac2 located in this last group have shown AHL acylase activity, whereas they are inactive on CGH substrates, β-lactam antibiotics, or bile salts [50]. Apart from *Au*AHLA, other Ntn-hydrolases from different groups have shown both penicillin and AHL acylase activity (Supplementary Material, Figure S3, enzymes with asterisk) such as AhlM [31], MacQ [45], PfmA from *Pseudoalteromonas flavipulchra* JG1 [51], *Kc*PGA from *Kluyvera citrophila* [52], *At*PVA from *Agrobacterium tumefaciens,* and *Pa*PVA from *Pectobacterium atrosepticum* [19]. Among these bifunctional enzymes, *Sl*PVA and *Au*AAC have also shown aculeacin A acylase activity [53].

Regarding substrate specificity, *Au*AHLA can hydrolyze different natural penicillins and several *N*-acyl-l-homoserine lactones, both aliphatic and 3-oxo substituted. The best catalytic efficiency has been determined for penicillin V, followed by C_12_-HSL. Surprisingly, catalytic efficiency using penicillin V as a substrate is 2.4-fold higher than C_12_-HSL and 7.3-fold higher than C_10_-HSL (Table 2). Kinetic parameters are drastically decreased using AHLs with 3-oxo substitution, and no catalytic activity was detected using C_4_-HSL, 3-oxo-C_6_-HSL, aculeacin A, and caspofungin as substrate. *Au*AHLA showed a catalytic efficiency for penicillin G 100-fold lower than for penicillin V, despite this enzyme is a heterodimer with an N-terminal catalytic Ser similar to PGAs and in contrast to PVAs, which are usually homotetrameric with a Cys at the N-terminal [49]. This unusual 3D structure has also been previously described for *Sm*PVA [30] and *Sl*PVA [29]. Catalytic efficiencies of *Au*AHLA using natural penicillins as substrate (Table 2) are significantly higher than those previously reported for *Au*AAC (34.79 and 4.55 mM^−1^ s^−1^ for penicillin K and V, respectively) [28] and *Sl*PVA (38.88, 21.85, and 0.075 mM^−1^ s^−1^ for penicillin V, dihydroF and G, respectively) [54]. Moreover, *Au*AHLA shows a catalytic efficiency for penicillin G similar to *Sm*PVA (2.1 mM^−1^ s^−1^), while it is significantly higher for penicillin V (51.9 mM^−1^ s^−1^) [30].

Comparable substrate specificity of *Au*AHLA has also been described for other acylases such as AhlM from *Streptomyces* sp. strain M664, which can degrade penicillin G and AHLs with acyl chains of eight carbons or more [31]. However, scarce kinetic data of AHL acylase activity have been reported, highlighting the studies with PvdQ and HacB from *P. aeruginosa* [55,56], *Kc*PGA [52], and penicillin V acylases from *Pectobacterium atrosepticum* (*Pa*PVA) and *Agrobacterium tumefaciens* (*At*PVA) [19]. It should be considered that kinetic parameters using a broad range of AHLs have been determined in this study, as well as previously described for *Sl*PVA and *Au*AAC [53], while a few AHLs have been used as substrate in other acylases kinetic studies.

Results observed in bioassay plate tests with *Chromobacterium violaceum* CV026 suggest the potential application of *Au*AHLA as a QQ agent. Similar results have been previously reported using *Sl*PVA and *Au*AAC [53], but the inhibitory effect on violacein production is larger and more evident using *Au*AHLA. The wide AHLs specificity of *Au*AHLA suggests that it could be an attractive enzyme for protein engineering to improve its properties as a quorum quenching agent.

Noteworthy, *Au*AHLA has efficiently synthesized penicillin V, confirming that this enzyme can carry out both acylation and deacylation of this substrate depending on the reaction conditions. Penicillin V synthetic parameters of *Au*AHLA can be compared with the observed for *Sl*PVA in similar reaction conditions [36]. In presence of 2.7% (*v*/*v*) DMSO, 0.3 IU/mL *Sl*PVA reaches a higher Y_max_ (94.5%) and higher S/H ratio (16.4). However, in presence of 40% (*v*/*v*) DMSO, 1 IU/mL *Sl*PVA reaches a Y_max_ of 81%, similar to 1 IU/mL *Au*AHLA in 32% (*v*/*v*) DMSO. Although the S/H ratio is lower for *Au*AHLA than *Sl*PVA in both conditions, the reaction time for Y_max_ of *Au*AHLA is shorter than *Sl*PVA. In addition, penicillin V synthesis by *Au*AHLA has reached a Y_max_ and S/H ratio higher than *Sm*PVA in similar conditions (66% and 3.2, respectively) [57].

Moreover, *Au*AHLA has catalyzed the enzymatic synthesis of natural AHLs such as C_6_-HSL, C_8_-HSL, C_10_-HSL, C_12_-HSL, 3-oxo-C_6_-HSL, 3-oxo-C_10_-HSL, and 3-oxo-C_12_-HSL, and the non-natural analog POHL, under the appropriate reaction conditions. Although enzymatic synthesis of POHL was slower than synthesis of penicillin V and needed a higher concentration of DMSO due to the low solubility of AHLs in water, both synthetic reactions reached a quite similar Y_max_. Currently, necessary AHLs for QS research are obtained by different strategies of chemical synthesis [14,58,59,60]. To date, only one example of enzymatic synthesis of aliphatic and hydroxylated AHLs has been described, using immobilized lipase from *Candida antarctica* (CAL) [61]. However, this study using the acylase *Au*AHLA is the first enzymatic synthesis report of 3-oxo-AHLs and non-natural aryl-HSLs. Enzymatic synthesis of AHLs and non-natural analogs could present several advantages over chemical synthesis, being a faster process with less solvent waste. Furthermore, biocatalysts are biocompatible, biodegradable, and non-toxic, enzymatic reactions are highly regio-, stereo-, and chemoselective, and they could be carried out at moderated temperature and pressure conditions [62,63].

As mentioned above, non-natural AHLs analogs can be used as QQ agents by blocking the QS receptor. In contrast to other QQ strategies, autoinducers analogs show some advantages, such as they are often highly diffusible through biological membranes, leading to a rapid QS inhibition that can be reversible or irreversible depending on the molecule used. Several substituted derivatives of POHL have been previously evaluated as QS receptor modulators, confirming their potential as QS antagonists. In particular, 4-NO_2_-POHL was a potent antagonist for the QS receptor TraR from *Agrobacterium tumefaciens* and 4-Br- and 4-I-POHL were antagonists for both receptors TraR from *A. tumefaciens* and LasR from *Pseudomonas aeruginosa* [37]. For this reason, it would be interesting to enzymatically synthesize POHL that could be used as a synthetic scaffold for obtaining these non-natural aryl-HSLs.

## 4. Materials and Methods

### 4.1. Chemical Reagents

Cell culture media were purchased from Becton-Dickinson (Franklin Lakes, NJ, USA); chemical reagents such as methanol, acetic acid and potassium phosphate from Fisher Scientific (Waltham, MA, USA); *N*-acyl-l-homoserine lactones (except *N*-(3-oxohexanoyl)-l-homoserine lactone, which was from Santa Cruz Biotechnology, Dallas, TX, USA), l-homoserine lactone hydrochloride (HSL), penicillin V, phenoxyacetic acid (POA), methyl phenoxyacetate (MPOA), methyl hexanoate, methyl decanoate, *p*-dimethylaminobenzaldehyde (PDAB), *o*-phthalaldehyde (OPA), kanamycin, and DMSO from Sigma-Aldrich (Burlington, MA, USA); methyl octanoate and methyl dodecanoate from Fluka (Burlington, MA, USA); methyl butyrate from Acros Organics (Waltham, MA, USA); methyl butyrylacetate from Alfa Aesar (Tewksbury, MA, USA); and methyl 3-oxodecanoate and methyl 3-oxododecanoate from Toronto Research Chemicals (Toronto, ON, Canada). 6-aminopenicillanic acid (6-APA) and natural aliphatic penicillins (penicillin K, penicillin F and penicillin dihydro-F) were provided by Antibióticos S.A. (León, Spain). All reagents used showed a purity level of at least ≥95%. Oligonucleotides were synthesized by Sigma-Aldrich (Burlington, MA, USA).

### 4.2. Microorganisms, Culture Conditions, Plasmids, and DNA Manipulation

Bacterial strains, plasmids, and oligonucleotides used are listed in Table 3. *Actinoplanes utahensis* NRRL 12052 was used as the DNA source [23], *Escherichia coli* DH5α was used as the host for subcloning experiments [64], and *Rhodococcus* sp. T104 KACC 21099 was used as the host for gene expression [38]. *A. utahensis* was cultured in S-YEME (yeast extract, malt extract, 0.5% glycine) liquid medium at 30 °C [65], *Rhodococcus* was grown in 2xYTG (yeast extract, bacto tryptone, NaCl, glucose) medium at 30 °C [38], and *E. coli* was cultured in Luria-Bertani (LB) medium at 37 °C [66]. *Chromobacterium violaceum* CV026 CECT 5999 was grown in LB agar plates at 30 °C for bioassays [67]. Bifunctional plasmids pEM4 [68] and pNV19 [69] were used to construct the *E. coli-Rhodococcus* shuttle vector pENV19 [39] for gene expression in *Rhodococcus* sp. T104. For that, the fragment of 227 bp containing the strong ermE* promoter and the MCS of the pEM4 vector was cloned between *Hind*III and *Eco*RI restriction site in pNV19 (Supplementary Material, Figure S4).

Plasmid purification was performed using the PureLink Quick Plasmid Miniprep Kit from Invitrogen (Waltham, MA, USA). Plasmidic DNA was sequenced according to Sanger [70] by BigDye Terminator v3.1 with an automatic DNA sequencer ABI Prism 3730 (Applied Biosystems Inc, Waltham, MA, USA) in Secugen S. L.

**Table 3:** Bacterial strains, plasmids, and primers ^#^ used.

Strain, Plasmid, or Primer	Relevant Description or Sequence	Reference
*Actinoplanes utahensis* NRRL 12052	Native *N*-acyl-homoserine lactone acylase producer	[23,24,71]
*Escherichia coli* DH5*α*	Host for recombinant plasmid F^−^ λ- φ80d*lac*ZΔM15 Δ(*lac*ZYA-*arg*F) U169 *rec*A1 *end*A1 *hsd*R17(r_K_^−^ m_K_^+^) *sup*E44 *thi*-1 *gyr*A96 *rel*A1	[64]
*Rhodococcus* sp. T104 KACC 21099	Host for gene expression (*Kn*^s^)	[38]
*Rhodococcus* pENV19*ahla*	Recombinant strain harboring the plasmid pENV19*ahla*	This study
*Chromobacterium violaceum* CV026 CECT 5999	cvil::mini-Tn5-mutant of *Chromobacterium violaceum* ATCC 31532 (Kn^R^)	[67]
pENV19	Shuttle vector for *E. coli*-*Rhodococcus* with the constitutive permE* promoter (5.1 Kb, *Kn*^R^ pAL5000ori permE * ColE1ori)	[29,38,39]
pENV19*ahla*	pENV19-containing *ahla* gene from *A. utahensis* NRRL 12052	This study
AHLA1	5’-GC**TCTAGA***GGAGG*TGCCGCCGTGGCCCGTCCGTTCA-3’	This study
AHLA2	5’-CG**GAATTC**CTCACCGCGGCGCTCGCTCGGTCAGTCTGAT-3’	This study

^#^ The restriction sites *Xba*I and *EcoR*I are shown in bold, ribosomal binding site sequence is shown in italics, and the start and the stop codons are shown underlined.

### 4.3. Overexpression of ahla Gene and Purification of Recombinant AuAHLA from Rhodococcus sp. T104

The hypothetical *ahla* gene (GenBank accession no. NZ_JRTT01000008.1), including its signal peptide coding sequence and the putative RBS sequence (GGAGG), was amplified by PCR using *Pfu*DNA polymerase (Thermo, Waltham, MA, USA) and genomic DNA from *A. utahensis* NRRL 12052 [23] as a template. PCR was performed in Mastercycler Personal equipment (Eppendorf, Hamburg, Germany), and amplification conditions were set according to the high G + C content of the actinomycetes genome [29,39]. *Xba*I and *EcoR*I restriction sites were included in the primers (Table 1) in order to subclone the PCR product in pENV19 vector. PCR products were purified by the commercial kit illustra GFX PCR DNA and Gel Band Purification (GE Healthcare, Chicago, IL, USA). Recombinant plasmid pENV19*ahla* was used to transform electrocompetent *Rhodococcus* sp. T104 cells [38].

To purify the recombinant *Au*AHLA (NCBI accession no. WP_052163432.1), the strain *Rhodococcus* pENV19*ahla* was cultivated in 2× YTG liquid medium with 100 μg/mL kanamycin at 30 °C for 72 h and 250 rpm orbital shaking. Cell-free supernatant was harvested by centrifugation at 3500× *g* and adjusted at pH 6.0. The enzyme was purified from the culture broth by cation-exchange chromatography on an SP-Sepharose Fast Flow column (Amersham Biosciences, Buckinghamshire, UK), equilibrated with 10 mM potassium phosphate pH 6.0 buffer. The enzyme was eluted with a linear gradient of NaCl (0–1 M) in the same buffer at a flow rate of 1 mL/min using an NGC Chromatography System (Bio-Rad, Hercules, CA, USA). The fractions showing acylase activity were pooled and concentrated by ultrafiltration, using an Amicon Ultra-15 filter (Millipore, Burlington, MA, USA). The purity of the protein was analyzed by 12.5% (*w*/*v*) SDS-PAGE, and protein concentration was determined according to Bradford [72].

### 4.4. Protein Sequence and Structure Analysis

To determine the N-terminal amino acid sequence, the protein bands after SDS-PAGE were transferred to a PVDF membrane Immobilon-P (Millipore, Burlington, MA, USA) [73] and sequenced by automatic Edman degradation in a Procise 494 Protein Sequencing System (Applied Biosystems Inc., Waltham, MA, USA) in the Protein Chemistry Facility (CIB-CSIC). The molecular weight and peptide fingerprinting analysis were performed on an Autoflex III MALDI-TOF/TOF instrument (Bruker Daltonics, Billerica, MA, USA) with a smart beam laser in the Proteomics and Genomics Facility (CIB-CSIC).

Circular dichroism (CD) spectra were recorded using a Jasco (Tokio, Japan) J-715 spectropolarimeter with a thermostated 0.1-cm-path-length quartz cell in the far-UV region. The CD readings were expressed as the mean residue molar ellipticity (deg cm^2^ dmol^−1^), and secondary structure information was obtained from CD spectra by using the CDNN V2.1 [74].

Analytical ultracentrifugation experiments were carried out with an Optima XLA ultracentrifuge (Beckman-Coulter, Brea, CA, USA) in the Molecular Interactions Facility (CIB-CSIC). Sedimentation coefficients (*S*) were obtained from the sedimentation velocity results, analyzed with SEDFIT version 16.1c software.

Transmission electron microscopy (TEM) analysis was performed in the Electron Microscopy Facility (CIB-CSIC). Micrographs were taken with a JEOL (Peabody, MA, USA) transmission electron microscope JEM-1230 operated at 100 kV and equipped with a digital camera CMOS TVIPS TemCam-F416 at nominal magnification of X5000. Protein samples were negatively stained with 1% (*w*/*v*) uranyl acetate on Formvar grids. Single images corresponding to *Au*AHLA particles were extracted using the EMAN program (developed by National Center for Macromolecular Imaging, Houston, TX, USA) [75].

### 4.5. Acylase Activity Assay

Acylase activity was routinely assayed, incubating 70 mM penicillin V in 0.3 M potassium phosphate pH 8.0 buffer at 45 °C for 20 min and 450 rpm. The enzymatic reaction was stopped by adding 20% (*v*/*v*) acetic acid, and 6-APA released was detected by adding 0.5% (*w*/*v*) PDAB reagent dissolved in methanol and measuring the absorbance at 405 nm [76]. All measurements were carried out in triplicate. One international activity unit (IU) was defined as the amount of enzyme-producing 1 µmol/min of 6-APA under the described assay conditions.

### 4.6. Biochemical Characterization and Kinetic Parameters

The optimum pH of purified *Au*AHLA was determined by assaying the activity at different pH values (4–11) in 20 mM citrate-phosphate-borate buffer with 220 mM constant ionic strength at 45 °C. The optimum temperature was also determined by assaying the activity at several temperatures (20–60 °C) in 0.3 M potassium phosphate pH 8.0 buffer. To determine pH stability, 0.1 µg of *Au*AHLA were incubated in 20 mM citrate-phosphate-borate buffer at pH values from 4 to 11, for 20 min at 4 °C. Residual acylase activity was assayed under optimal conditions, as described above. Thermal stability was also studied by incubating 0.1 µg of *Au*AHLA at various temperatures (20–60 °C) for 20 min in 0.3 M potassium phosphate pH 8.0 buffer. After 10 min in an ice bath, residual acylase activity was measured at 45 °C as described above. All assays were carried out in triplicate.

Kinetic parameters of *Au*AHLA were determined using the following penicillins and l-enantiopure AHLs: penicillin V, penicillin K, penicillin dihydroF, penicillin G, *N*-butanoyl-l-homoserine lactone (C_4_-HSL), *N*-hexanoyl-l-homoserine lactone (C_6_-HSL), *N*-octanoyl-l-homoserine lactone (C_8_-HSL), *N*-decanoyl-l-homoserine lactone (C_10_-HSL), *N*-dodecanoyl-l-homoserine lactone (C_12_-HSL), *N*-(3-oxohexanoyl)-l-homoserine lactone (3-oxo-C_6_-HSL), *N*-(3-oxooctanoyl)-l-homoserine lactone (3-oxo-C_8_-HSL), *N*-(3-oxodecanoyl)-l-homoserine lactone (3-oxo-C_10_-HSL), and *N*-(3-oxododecanoyl)-l-homoserine lactone (3-oxo-C_12_-HSL). Enzymatic reactions containing 0.18 µg of *Au*AHLA, 20 µL of increasing concentrations of penicillins (dissolved in water) or AHLs (dissolved in DMSO) and 0.2 M potassium phosphate pH 8.0 buffer (final volume of 100 µL), were incubated at 45 °C for 10 min and 450 rpm. Primary amines released during the enzymatic reactions were determined by the fluorometric assay according to Velasco-Bucheli et al. [53]. The enzymatic reaction was mixed with OPA reagent, and fluorescence intensity was determined employing a Thermo Varioskan LUX (Thermo, Waltham, MA, USA) with 355 nm and 460 nm as excitation and emission wavelengths, respectively. All enzymatic reactions were carried out in triplicate. Kinetics curves were fitted to equation: *v* = (V_max_ × S)/(K_m_ + S), and kinetic constants were determined by nonlinear regression using Hyper32 program (available online: http://homepage.ntlworld.com/john.easterby/hyper32.html, accessed on January 2020).

### 4.7. Enzymatic Synthesis of Penicillin V and AHLs

Kinetically controlled synthesis of penicillin V by *Au*AHLA was carried out according to Hormigo et al. [36] with slight modifications. The reaction mixture containing 1 IU/mL *Au*AHLA, 100 mM 6-APA, 20 mM MPOA and 3% or 32% (*v*/*v*) DMSO, was incubated in 100 mM potassium phosphate pH 7.0 buffer at 30 °C and 800 rpm. At different incubation times, 20 µL of the enzymatic reaction were mixed with 20 µL of cold methanol and analyzed by HPLC using an Agilent 1100 equipment (Santa Clara, CA, USA) and a Phenomenex (Torrance, CA, USA) Luna column C18(2), 250 × 4.6 mm (5 μM particle size); with a 65% (*v*/*v*) methanol solution containing 0.05% (*v*/*v*) phosphoric acid as mobile phase. The flow rate was fixed at 0.8 mL/min, and the UV detector was set at 260 nm. Retention times of substrates and products were 4.0 (6-APA), 6.6 (POA), 9.6 (MPOA), and 11.3 min (penicillin V). Concentrations of penicillin V, POA, and MPOA were estimated with calibration curves using standard solutions. All determinations were carried out in triplicate. Synthetic activity (V_S_) was established as the initial rate of synthesis (penicillin V production per unit time, expressed as mM/h), whereas hydrolytic activity (V_h_) was established as the initial rate of hydrolysis of MPOA (POA production per unit time, expressed as mM/h). The ratio of synthetic activity to hydrolytic activity (S/H ratio) was calculated as V_S_/V_h_.

Enzymatic synthesis of AHLs were carried out with 2 IU/mL *Au*AHLA, 100 mM L-HSL, 20 mM acyl donor (methyl butyrate, hexanoate, octanoate, decanoate, dodecanoate, butyrylacetate, 3-oxodecanoate, or 3-oxododecanoate), and 23% or 50% (*v*/*v*) DMSO in 100 mM potassium phosphate pH 7.0 buffer. These mixtures were incubated at 30 °C and 800 rpm for 24 h, and then the samples were evaporated at 45 °C to dryness using a speed vacuum concentrator Thermo Scientific SPD121P (Waltham, MA, USA), stored at −20 °C, and dissolved in methanol prior to analysis. Enzymatic synthesized AHLs were analyzed by LC-MS/MS using a Shimadzu LCMS8030 (Kyoto, Japan) equipment and a Phenomenex (Torrance, CA, USA) Gemini Column C18 110 Å (150 × 2 mm 5 μm particle size) with a Phenomenex C18 pre-column (4 × 2 mm) in the Mass Spectrometry Facility (Faculty of Chemistry, UCM). Mobile phase A was water and mobile phase B was methanol, and the gradient profile was as follows: 0% phase B for 1 min, from 0% to 100% phase B for 5 min, 100% phase B for 1.5 min, from 100% to 0% phase B for 1 min and 0% phase B for 1.5 min at a flow rate of 0.4 mL/min. Analysis of AHLs was performed by multiple reaction monitoring (MRM) mode to monitor de parent ion-product ion (*m*/*z*) of the analyte, and mass transitions of each molecule are listed in Supplementary Material, Table S3.

### 4.8. Bioassay Activity of Hydrolyzed and Synthetized AHLs Using Chromobacterium violaceum CV026

Bioactive AHLs hydrolyzed by *Au*AHLA were tested using *C. violaceum* CV026 [53,67]. Hydrolysis reactions of different AHLs were carried out in 50 µL containing 1 mM of the corresponding AHL, 0.5 IU/mL *Au*AHLA, 100 mM potassium phosphate pH 8.0 buffer and 10% (*v*/*v*) DMSO and were incubated at 45 °C for 2 h and 450 rpm. Then, these mixtures were applied in wells punched in LB agar medium with preinduced *C. violaceum* CV026 and incubated at 30 °C for 24 h (forward bioassay). For enzymatic reactions using AHLs with acyl chains longer than C_8_-HSL, the reverse bioassay was performed, adding 5 μM C_6_-HSL to the agar plates together with *C. violaceum* CV026 [53]. Similarly, the synthesis of bioactive AHLs was tested using an aliquot of 50 μL of each reaction mixture after 24 h at 30 °C following the protocol described above.

## 5. Conclusions

*Au*AHLA can hydrolyze a broad range of substrates, including different natural penicillins and several *N*-acyl-l-homoserine lactones, both aliphatic and 3-oxo substituted. Moreover, this enzyme shows quorum quenching activity since it inhibits the production of violacein by *C. violaceum* CV026.

*Au*AHLA is the first reported AHL acylase capable of synthesizing different bioactive *N*-acyl-l-homoserine lactones, both aliphatic and 3-oxo substituted, using L-HSL and methyl fatty acids as substrates. Enzymatic synthesis of AHLs arises as an alternative method that might be faster, cheaper, and eco-friendlier than the organic synthesis of these compounds. Furthermore, this enzymatic method could allow obtaining non-natural analogs of AHLs to block QS receptors and inhibit the signal of autoinducers.

## Figures and Tables

**Table 1:** Kinetic parameters of AuAHLA using different natural penicillins and AHLs as substrate.

Substrate	*Km* (mM)	*kcat* (s^−1^)	*kcat/Km* (mM^−1^ s^−1^)
Penicillin V	0.472 ± 0.05	106.34 ± 2.84	239.3
Penicillin dihidroF	0.199 ± 0.02	9.15 ± 0.28	48.8
Penicillin K	0.675 ± 0.19	2.33 ± 0.23	3.66
Penicillin G	1.21 ± 0.30	2.71 ± 0.18	2.39
C_4_-HSL	ND	ND	ND
C_6_-HSL	2.25 ± 0.50	6.07 ± 0.69	2.87
C_8_-HSL	0.860 ± 0.15	3.52 ± 0.29	4.35
C_10_-HSL	0.084 ± 0.021	2.59 ± 0.23	32.7
C_12_-HSL	0.008 ± 0.002	0.75 ± 0.045	99.5
3-oxo-C_6_-HSL	ND	ND	ND
3-oxo-C_8_-HSL	1.04 ± 0.42	0.19 ± 0.028	0.20
3-oxo-C_10_-HSL	0.238 ± 0.086	0.50 ± 0.097	2.22
3-oxo-C_12_-HSL	0.092 ± 0.021	0.53 ± 0.039	6.11

Reaction conditions: 45 °C, 0.2 M potassium phosphate pH 8.0 buffer, 0.18 μg *Au*AHLA and 20% (*v*/*v*) DMSO in the case of AHLs. ND: no activity detected.

**Table 2:** Parameters for the kinetically controlled synthesis of penicillin V and POHL catalyzed by *Au*AHLA.

Product	*Au*AHLA (IU/mL)	DMSO (%)	V_s_ (mM/h)	V_h_ (mM/h)	S/H	Y_max_ (%)	Time for Y_max_ (h)
Penicillin V	0.125	3	0.85	0.40	2.13	30.8	24
	0.25	3	1.57	0.53	2.99	50.5	24
	1	3	3.81	0.55	6.94	72.6	6
	1	32	3.70	0.37	10.1	76.8	8
POHL	1	23	1.35	0.23	5.87	46.4	24
	1	50	1.11	0.08	13.1	69.9	24

Reaction conditions: 30 °C, 0.1 M potassium phosphate pH 7.0 buffer, 100 mM acyl acceptor (6-APA or HSL) and 20 mM MPOA.

## Data Availability

The data presented in this study are fully available in the main text and Supplementary Material of this article.

## Supplementary Materials

The following are available online at https://www.mdpi.com/article/10.3390/antibiotics10080922/s1, Figure S1: Multiple amino acid sequence alignment of *Au*AHLA and other acylases from the Ntn-hydrolase superfamily by ClustalW. Figure S2: Purified *Au*AHLA analysis. Figure S3: Phylogenetic analysis of *Au*AHLA and other acylases from the Ntn-hydrolase superfamily. Figure S4: Shuttle vector pENV19 construction. Table S1: Amino acids involved in the catalysis predicted by COBALT sequence alignment using *Au*AHLA, *Sl*PVA, *Au*AAC, and other 174 acylases and their characteristics. Table S2: Purification table of *Au*AHLA by ionic exchange chromatography. Table S3: MRM parameters of AHLs analysis.
