# Supplementary material for: Novel Bifunctional Acylase from Actinoplanes utahensis: A Versatile Enzyme to Synthesize Antimicrobial Compounds and Use in Quorum Quenching Processes
Source: Antibiotics (Basel). 2021 Jul 29;10(8):922. doi: 10.3390/antibiotics10080922 (PMC8388760; doi:10.3390/antibiotics10080922)
Supplement: Supplementary file 1 [file antibiotics-10-00922-s001.zip › antibiotics-1308288-supplementary.pdf]

**Supplementary material**

# Novel Bifunctional Acylase from *Actinoplanes utahensis*: a Versatile Enzyme to Synthesize Antimicrobial Compounds and Use in Quorum Quenching Processes

**Lara Serrano-Aguirre[1], Rodrigo Velasco-Bucheli[1,#], Begoña García-Álvarez[1], Ana Saborido[1], Miguel Arroyo[1], Isabel de la Mata[1,*]**

[1] Department of Biochemistry and Molecular Biology, Faculty of Biology, Universidad Complutense de Madrid (UCM), José Antonio Nováis 12, E-28040 Madrid, Spain. E-mail addresses: larase01@ucm.es (L.S-A), rvelasc@unal.edu.co (R.V-B.); begoga01@ucm.es (B.G-A), asaborid@ucm.es (A.S.), marroyos@ucm.es (M.A.), idlmata@ucm.es (I.D.L.M.).

* Correspondence: idlmata@ucm.es; Tel.: +34-913944150

[#] Present address: Roche Diagnostics GmbH, Penzberg, Germany

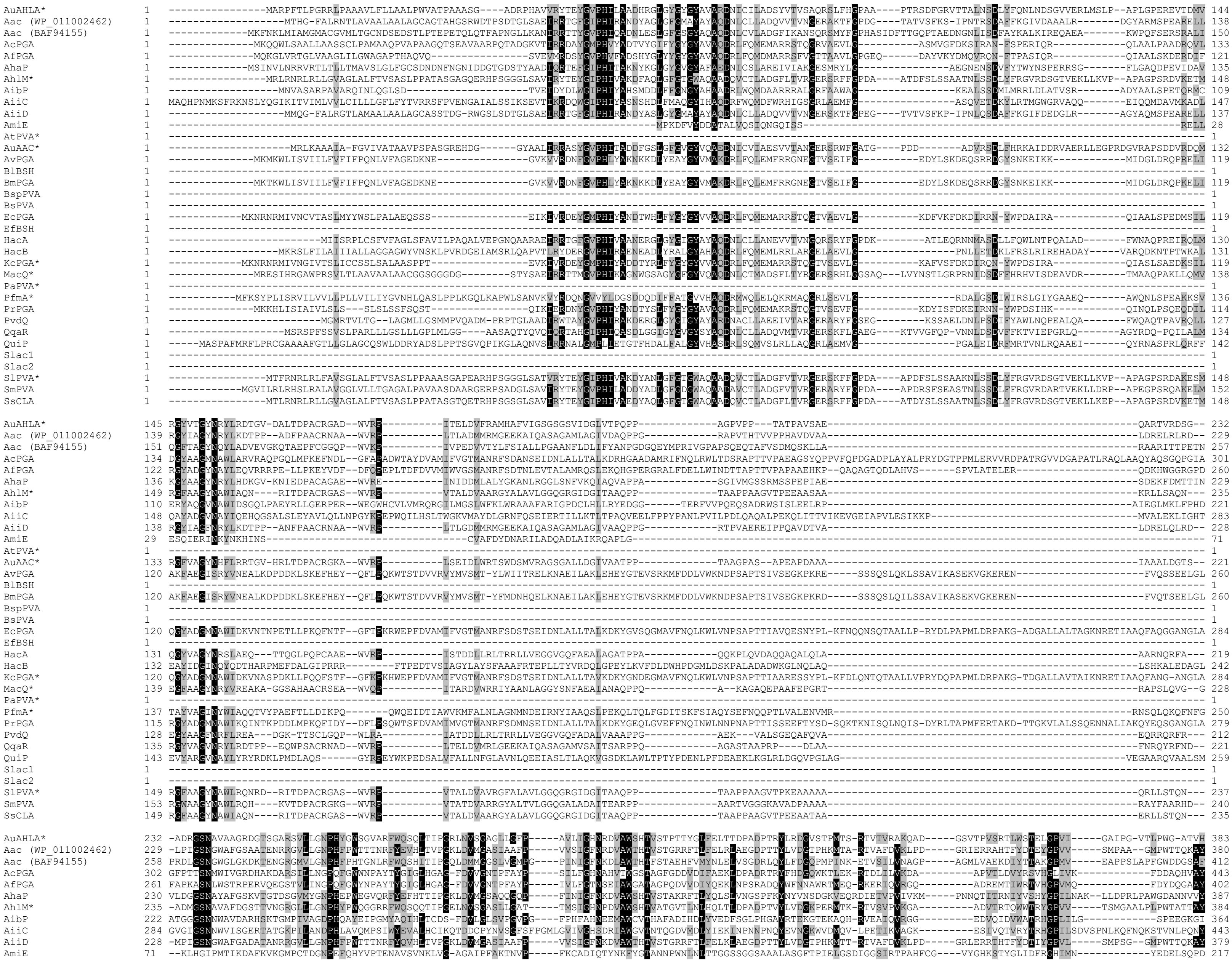
AuAHLA*
Aac (WP_011002462)
Aac (BAF94155)
AcPGA
AfPGA
AhaP
AhlM*
AibP
AiiC
AiiD
AmiE
AtPVA*
AuAAC*
AvPGA
BlBSH
BmPGA
BspPVA
BsPVA
EcPGA
EfBSH
HacA
HacB
KcPGA*
MacQ*
PaPVA*
PfmA*
PrPGA
PvdQ
QqaR
QuiP
Slac1
Slac2
SlPVA*
SmPVA
SsCLA

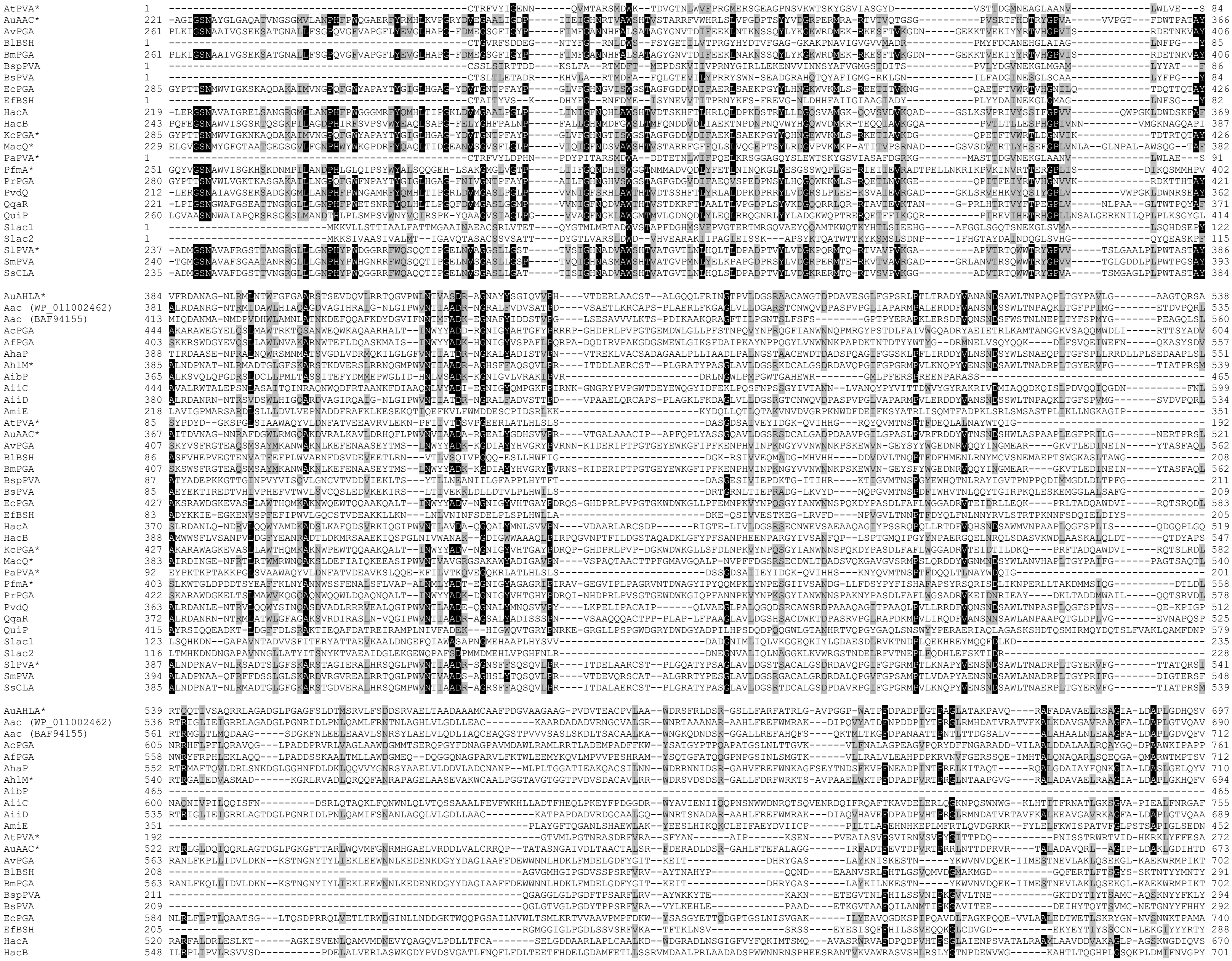
AtPVA*
AuAAC*
AvPGA
BlBSH
BmPGA
BspPVA
BsPVA
EcPGA
EfBSH
HacA
HacB
KcPGA*
MacQ*
PaPVA*
PfmA*
PrPGA
PvdQ
QqaR
QuiP
Slac1
Slac2
SlPVA*
SmPVA
SsCLA
AuAHLA*
Aac (WP_011002462)
Aac (BAF94155)
AcPGA
AfPGA
AhaP
AhlM*
AibP
AiiC
AiiD
AmiE

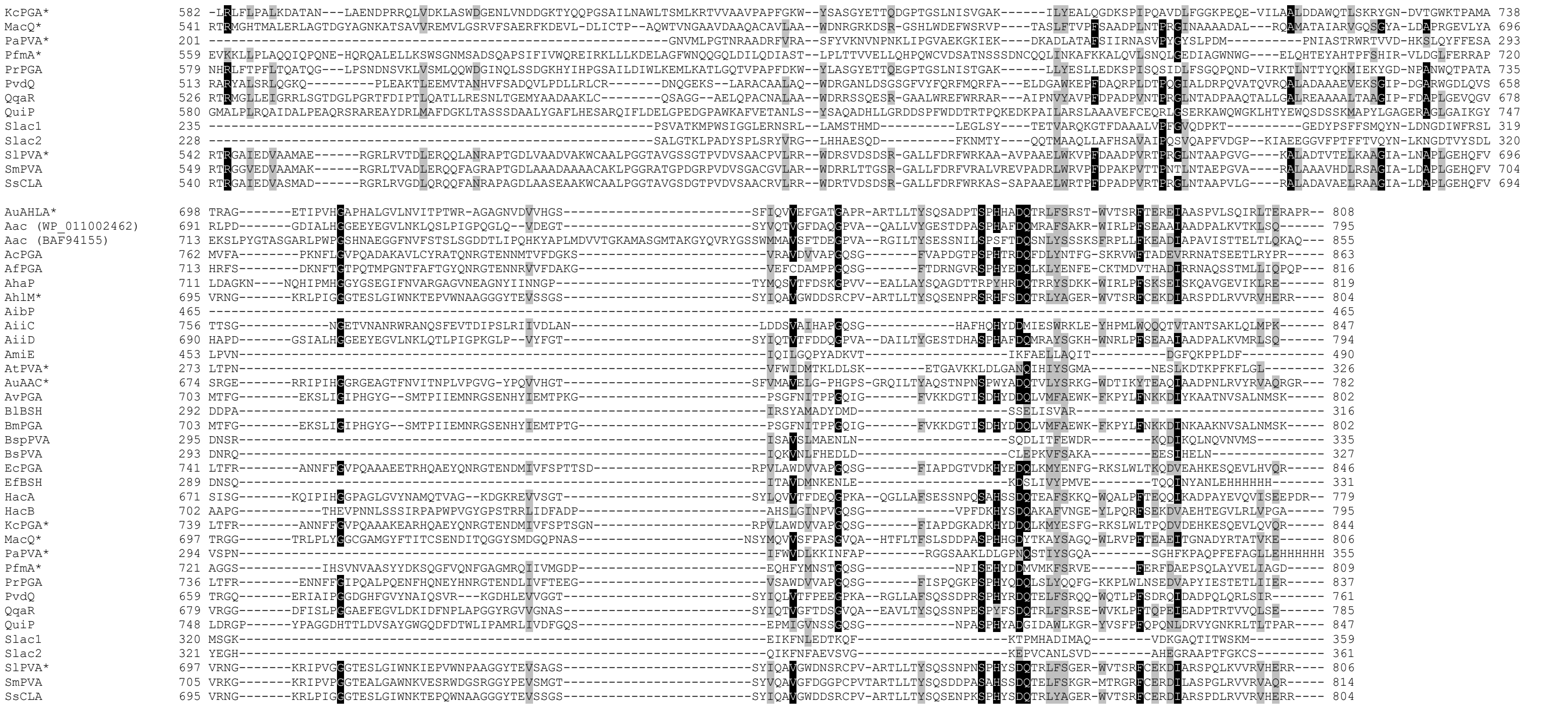

**Supplementary Figure S1.** Multiple amino acid sequence alignment of *Au*AHLA and other acylases from the Ntn-hydrolase superfamily by ClustalW. Ntn-hydrolases with reported AHL acylase activity: AiiD, from *Ralstonia* sp. XJ12B (NCBI accession no. AAO41113); PvdQ, from *Pseudomonas aeruginosa* PAO1 (AAG05773); AhlM, from *Streptomyces* sp. M664 (AAT68473); QuiP, from *P. aeruginosa* PAO1 (AAG04421); HacA, from *Pseudomonas syringae* B728a (AAY37014); HacB, from *P. syringae* B728a (AAY39885); AiiC, from *Anabaena* sp. strain PCC7120 (BAB75623); Aac, from *Shewanella* sp. Strain MIB015 (BAF94155); QqaR, from *Deinococcus radiodurans* R1 (WP_010889514); MacQ, from *Acidovorax* sp. MR-S7 (BAV56778), PfmA, *Pseudoalteromonas flavipulchra* JG1 (ASS36259); AmiE, *Acinetobacter* sp. Ooi24 (BAP18758); AibP, from *Brucella melitensis* (AAL53453); Aac, from *Ralstonia solanacearum* GMI1000 (WP_011002462); *Sl*PVA, from *Streptomyces lavendulae* ATCC 13664 (AAU09670), *Au*AAC, from *Actinoplanes utahensis* NRRL 12052 (WP_043523659); AhaP from *Psychrobacter* sp. M9-54-1 (WP_200663734.1); *S*lac1, from *Shewanella loihica* PV-4 (ABO22553.1); *S*lac2, from *Shewanella loihica* PV-4 (ABO24671.1); and *Au*AHLA, from *A. utahensis* NRRL 12052 (WP_052163432). Ntn-hydrolases with reported penicillin G acylase activity: *Ec*PGA, from *Escherichia coli* ATCC 11105 (P06875); *Kc*PGA, from *Kluyvera citrophila* DSM 2660 (P07941); *Ac*PGA, from *Achromobacter* sp. strain CCM 4824 (AAY25991); *Bm*PGA, from *Bacillus megaterium* ATCC 14945 (Q60136); *Af*PGA, from *Alcaligenes faecalis* ATCC 19018 (AAB71221); *Av*PGA, from *Arthrobacter viscosus* ATCC 15294 (P31956); and *Pr*PGA, from *Providencia rettgeri* (AAP86197). Ntn-hydrolases with reported penicillin V acylase activity: *Sm*PVA, from *Streptomyces mobaraensis* (BAF51977); *At*PVA, from *Agrobacterium tumefaciens* (5J9R); *Pa*PVA, from *Pectobacterium atrosepticum* (4WL2);

*Bsp*PVA, from *Bacillus sphaericus* (3PVA); and *Bs*PVA, from *Bacillus subtilis* (2OQC). Ntn-hydrolases with reported bile salt hydrolase activity: *Bl*BSH, from *Bifidobacterium longum* (2HF0), and *Ef*BSH, from *Enterococcus feacalis* (4WL3). Ntn-hydrolases with reported cyclic lipopeptide acylase activity were as follows: *Ss*CLA, from *Streptomyces* sp. FERM-BP5809 (BAD07025). Bifunctional acylases (with both penicillin and AHL acylase activities) are indicated by asterisks and, among them, *Sl*PVA and *Au*AAC present aculeacin A acylase activity as well.

**Supplementary Table S1.** Amino acids involved in the catalysis predicted by COBALT sequence alignment using *Au*AHLA, *Sl*PVA, *Au*AAC and other 174 acylases, and their characteristics*.

| Subunit | Residue | | | Secondary structure | Conserved | | Most common | |
|---|---|---|---|---|---|---|---|---|
| | ***Sl*PVA** | ***Au*AAC** | **Residue** | | **Residue** | **Side chain** | **Residue** | **Side chain** |
| β (catalytic residues) | βSer1 | βSer1 | βSer1 | Coil | S (100 %) | Polar (100 %) | S (100 %) | Polar (100 %) |
| | βHis23 | βHis23 | βHis23 | Coil | H (84 %) | Basic (87 %) | H (84 %) | Basic (87 %) |
| | βVal70 | βVal70 | βVal70 | Coil | V (33 %) | Nonpolar (63 %) | V (33 %) | Nonpolar (63 %) |
| | βAsn272 | βAsn268 | βAsn274 | Coil | N (99 %) | Polar (100 %) | N (99 %) | Polar (100 %) |
| α (substrate binding pocket) | αAla154 | αMet149 | αVal157 | Helix | A (22 %)<br>M (6 %)<br>V (12 %) | Nonpolar (66 %)<br>Nonpolar (66 %)<br>Nonpolar (66 %) | A (22 %) | Nonpolar (66 %) |
| | αGly158 | αGly153 | αGly161 | Coil | G (34 %) | Polar (55 %) | G (34 %) | Polar (55 %) |
| | αThr167 | αVal162 | αVal170 | Coil[*Sl*PVA]<br>Helix[*Au*AAC, *Au*AHLA] | T (9 %)<br>V (10 %) | Polar (25 %)<br>Nonpolar (60 %) | F (14 %) | Nonpolar (60 %) |
| β (substrate binding pocket) | βGly8 | βAla8 | βArg8 | Coil | G (28 %)<br>A (5 %)<br>R (4 %) | Polar (36 %)<br>Nonpolar (47 %)<br>Basic (17 %) | P (41 %) | Nonpolar (47 %) |
| | βThr10 | βAla10 | βGly10 | Coil | T (2 %)<br>A (5 %)<br>G (2 %) | Polar (8 %)<br>Nonpolar (13 %)<br>Polar (8 %) | K (41 %) | Basic (80 %) |
| | βTyr24 | βPhe24 | βTyr24 | Coil | Y (4 %)<br>F (24 %) | Polar (12 %)<br>Nonpolar (77 %) | L (43 %) | Nonpolar (77 %) |
| | βArg31 | βArg31 | βArg31 | Helix[*Sl*PVA]<br>Strand[*Au*AAC, *Au*AHLA] | R (24 %) | Basic (24 %) | R (24 %) | Nonpolar (53 %) |
| | βPhe32 | βPhe32 | βPhe32 | Helix[*Sl*PVA]<br>Strand[*Au*AAC, *Au*AHLA] | F (24 %) | Nonpolar (72 %) | W (42 %) | Nonpolar (72 %) |
| | βTrp33 | βTyr33 | βTrp33 | Helix[*Sl*PVA]<br>Strand[*Au*AAC, *Au*AHLA] | W (14 %)<br>Y (62 %) | Nonpolar (31 %)<br>Polar (69 %) | Y (62 %) | Polar (69 %) |
| | βLeu50 | βLeu50 | βLeu50 | Coil | L (40 %) | Nonpolar (86 %) | L (40 %) | Nonpolar (86 %) |
| | βSer53 | βAsp53 | βPhe53 | Coil | S (6 %)<br>D (1 %)<br>F (2 %) | Polar (25 %)<br>Acidic (1 %)<br>Nonpolar (75 %) | L (27 %) | Nonpolar (75 %) |
| | βSer57 | βGlu57 | βLeu57 | Strand | S (9 %)<br>E (1 %)<br>L (18 %) | Polar (26 %)<br>Acidic (1 %)<br>Nonpolar (59 %) | V (21 %) | Nonpolar (59 %) |
| | βIle58 | βIle58 | βIle58 | Strand | I (40 %) | Nonpolar (93%) | I (40 %) | Nonpolar (93 %) |
| | βSer67 | βSer67 | βSer67 | Strand | S (20 %) | Polar (98 %) | G (38 %) | Polar (98 %) |
| | βHis68 | βHis68 | βHis68 | Strand | H (26 %) | Basic (26 %) | H (26 %) | Nonpolar (49 %) |
| | βThr69 | βThr69 | βThr69 | Coil | T (94 %) | Polar (99 %) | T (94 %) | Polar (99 %) |
| | βThr72 | βThr72 | βThr72 | Helix | T (23 %) | Polar (54 %) | T (23 %) | Polar (54 %) |
| | βVal186 | βVal182 | βLeu188 | Strand | V (16 %)<br>L (15 %) | Nonpolar (54 %)<br>Nonpolar (54 %) | Q (20 %) | Nonpolar (54 %) |

*Secondary structure of each residue, percentage of conserved amino acids and most common residue on each position and side chain properties.

**(A)**

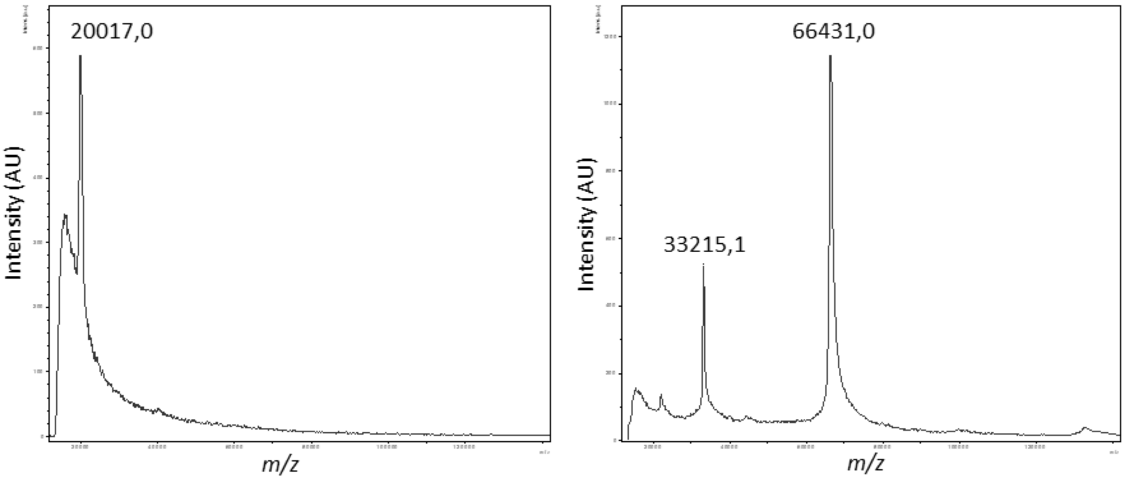


**(B)**

```
MARPFTLPGR RLPAAAVLFL LAALPWVATP AAASGADRPH AVVRYTEYGV PHILAADHRG  60
LGYGYGYAVA RDNICILADS YVTVSAQRSL FHGPAAPTRS DFGRVTTALN SDLYFQNLND 120
SGVVERLMSL PAPLGPEREV TDMVRGYVTG YNRYLRDTGV DALTDPACRG ADWVRPITEL 180
DVFRAMHAFV IGSGSGSVID GLVTPQPPAG PVPPTATPAV SAEQARTVRD SGADRGSNAV 240
AAGRDGTSGA RSVLLGNPHY GWSGVARFWQ SQLTIPGRLN VSGAGLLGFP AVLIGHNRDV 300
AWSHTVSTPT TYGLFELTTD PADPTRYLRD GVSTPMTSRT VTVRAKQADG SVTPVSRTLW 360
STELGPVIGA IPGVTLPWGA TVHVFRDANA GNLRMLNTWF GFGAARSTSE VDQVLRRTQG 420
VPWLNTVASD RAGNAYYSGI QVVPHVTDER LAACSTALGQ QLFRINGTPV LDGSRAACAW 480
GTDPDAVESG LFGPSRLPTL TRADYVANAN DSAWLTNPAQ PLTGYPAVLG AAGTQRSART 540
QQTIVSAQRR LAGADGLPGA GFSLDTMSRV LFSDDSRVAE LTAADAAAMC AAFPDGVAAG 600
AAGPVDVTEA CPVLAAWDRS FRLDSRGSLL FARFATRLGA VPGGPWATPF DPADPIGTPA 660
GLATAKPAVQ RAFADAVAEL RSAGIALDAP LGDHQSVTRA GETIPVHGAP HALGVLNVIT 720
PTWRAGAGNV DVVHGSSFIQ VVEFGATGAP RARTLLTYSQ SADPTSPHHA DQTRLFSRST 780
WVTSRFTERE IAASPVLSQI RLTERAPR                                   808
```

**(C)**

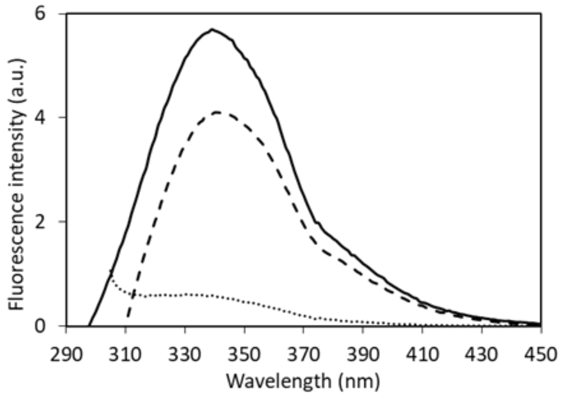


**(D)**

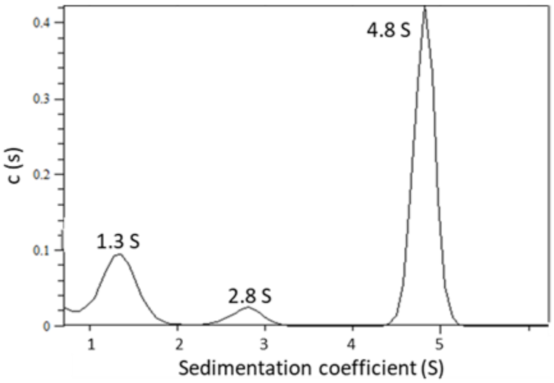


**Supplementary Figure S2.** Purified *Au*AHLA analysis. **(A)** MALDI-TOF analysis of $\alpha$-subunit (left) and β-subunit (right) of *Au*AHLA. **(B)** Mass fingerprint of $\alpha$-subunit (highlighted in grey) and β-subunit (highlighted in black) of *Au*AHLA. **(C)** Fluorescence emission spectra of *Au*AHLA: $\lambda_{ex}$ = 275 nm (solid line), $\lambda_{ex}$ = 295 nm (dashed line) and contribution of tyrosine residues to the emission spectra (dotted line). **(D)** Analytical ultracentrifugation analysis of *Au*AHLA: c(s) distribution as a function of sedimentation coefficient (S).

**Supplementary Table S2.** Purification table of *Au*AHLA by ionic exchange chromatography.

| Step | Activity (IU/mL) | Protein concentration (mg/mL) | Specific activity (IU/mg) | Yield (%) | Purification (fold) |
|---|---|---|---|---|---|
| **Cell-free broth** | 0.19 | 0.042 | 4.5 | 100 | 1 |
| **SP Sepharose** | 0.60 | 0.011 | 54.5 | 37 | 12 |
| **Ultrafiltration** | 8.40 | 0.096 | 87.5 | 22 | 19 |

**Supplementary Table S3.** MRM parameters of AHLs analysis.

| AHLs | Retention time (min) | Parent ion (*m*/*z*) | Product ions (*m*/*z*) | Collision Energy (V) |
|---|---|---|---|---|
| | | | 43.05 | -27 |
| **$C_4$-HSL** | 2.9 | 172.10 | 71.00 | -12 |
| | | | 102.05 | -14 |
| | | | 42.95 | -30 |
| **$C_6$-HSL** | 4.7 | 200.10 | 98.95 | -12 |
| | | | 102.05 | -12 |
| | | | 210.20 | -15 |
| **$C_8$-HSL** | 5.7 | 228.10 | 109.10 | -15 |
| | | | 102.10 | -15 |
| | | | 238.30 | -17 |
| **$C_{10}$-HSL** | 6.2 | 256.10 | 102.10 | -17 |
| | | | 109.10 | -17 |
| | | | 266.20 | -17 |
| **$C_{12}$-HSL** | 6.6 | 284.10 | 109.10 | -17 |
| | | | 102.10 | -17 |
| | | | 85.10 | 18 |
| **3-oxo-$C_6$-HSL** | 3.4 | 212.10 | 87.05 | 10 |
| | | | 57.05 | 30 |
| | | | 143.15 | 13 |
| **3-oxo-$C_{10}$-HSL** | 5.8 | 268.10 | 141.20 | 20 |
| | | | 57.05 | 35 |
| | | | 171.20 | 14 |
| **3-oxo-$C_{12}$-HSL** | 6.3 | 296.30 | 169.20 | 17 |
| | | | 57.10 | 11 |

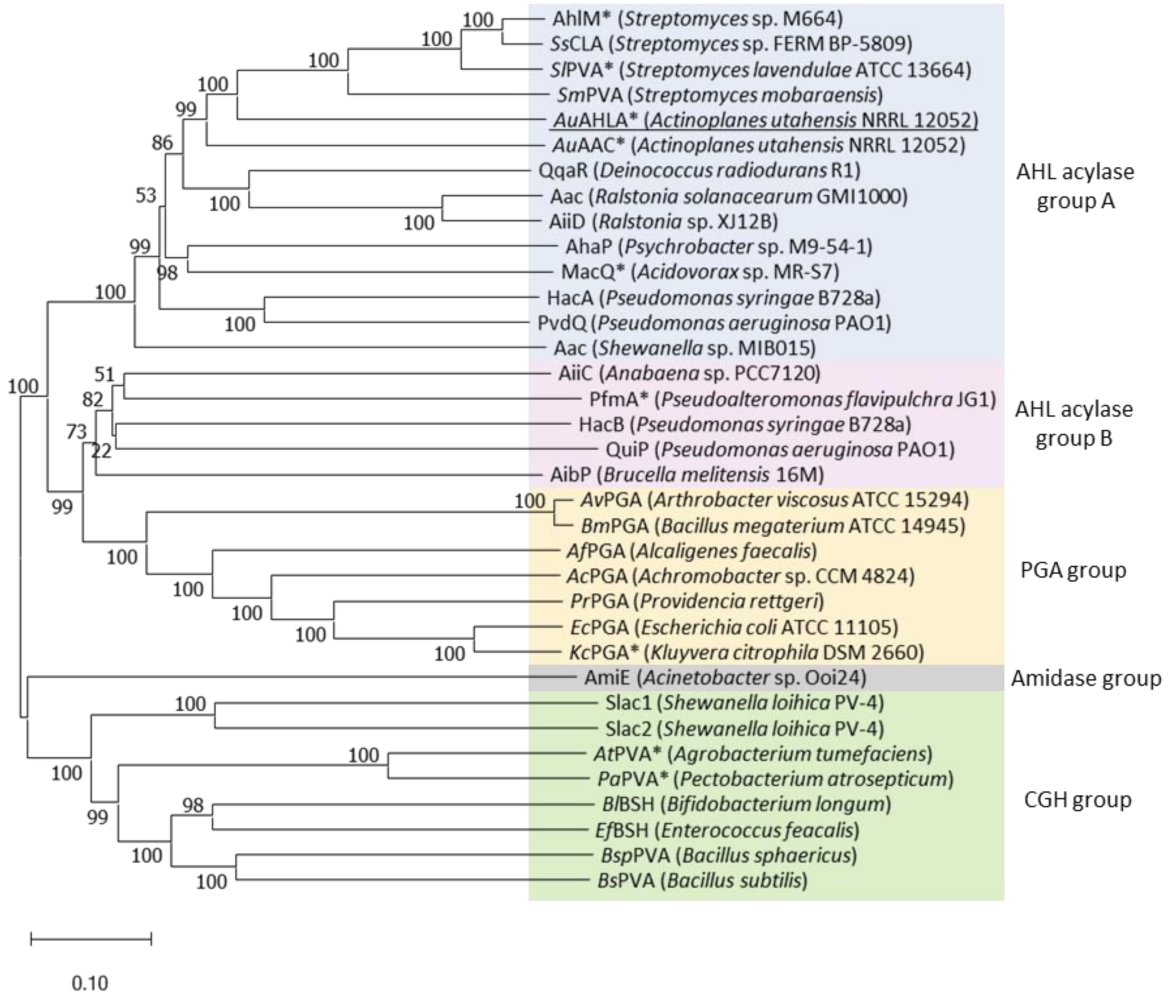


**Supplementary Figure S3**. Phylogenetic analysis of *Au*AHLA and other acylases from the Ntn-hydrolase superfamily. Evolutionary analysis was conducted in MEGA X and the phylogenetic tree was inferred using the Neighbor-Joining method. The percentage of replicate trees in which the associated taxa clustered together in the bootstrap test (1000 replicates) are shown next to the branches. The evolutionary distances were computed using the p-distance method and are in the units of the number of amino acid differences per site. Ntn-hydrolases with reported AHL acylase activity: AiiD, from *Ralstonia* sp. XJ12B (NCBI accession no. AAO41113); PvdQ, from *Pseudomonas aeruginosa* PAO1 (AAG05773); AhlM, from *Streptomyces* sp. M664 (AAT68473); QuiP, from *P. aeruginosa* PAO1 (AAG04421); HacA, from *Pseudomonas syringae* B728a (AAY37014); HacB, from *P. syringae* B728a (AAY39885); AiiC, from *Anabaena* sp. strain PCC7120 (BAB75623); Aac, from *Shewanella* sp. Strain MIB015 (BAF94155); QqaR, from *Deinococcus radiodurans* R1 (WP_010889514); MacQ, from *Acidovorax* sp. MR-S7 (BAV56778), PfmA, from *Pseudoalteromonas flavipulchra* JG1 (ASS36259); AmiE, *Acinetobacter* sp. Ooi24 (BAP18758); AibP, from *Brucella melitensis* (AAL53453); Aac, from *Ralstonia solanacearum* GMI1000 (WP_011002462); *Sl*PVA, from *Streptomyces lavendulae* ATCC 13664 (AAU09670), *Au*AAC, from *Actinoplanes utahensis* NRRL 12052 (WP_043523659); AhaP, from *Psychrobacter* sp. M9-54-1 (WP_200663734.1); *Sl*ac1, from *Shewanella loihica* PV-4 (ABO22553.1); *Sl*ac2, from *Shewanella loihica* PV-4 (ABO24671.1); and *Au*AHLA, from *A. utahensis* NRRL 12052 (WP_052163432). Ntn-hydrolases with reported penicillin G acylase activity: *Ec*PGA, from

*Escherichia coli* ATCC 11105 (P06875); *Kc*PGA, from *Kluyvera citrophila* DSM 2660 (P07941); *Ac*PGA, from *Achromobacter* sp. strain CCM 4824 (AAY25991); *Bm*PGA, from *Bacillus megaterium* ATCC 14945 (Q60136); *Af*PGA, from *Alcaligenes faecalis* ATCC 19018 (AAB71221); *Av*PGA, from *Arthrobacter viscosus* ATCC 15294 (P31956); and *Pr*PGA, from *Providencia rettgeri* (AAP86197). Ntn-hydrolases with reported penicillin V acylase activity: *Sm*PVA, from *Streptomyces mobaraensis* (BAF51977); *At*PVA, from *Agrobacterium tumefaciens* (5J9R); *Pa*PVA, from *Pectobacterium atrosepticum* (4WL2); *Bsp*PVA, from *Bacillus sphaericus* (3PVA); and *Bs*PVA, from *Bacillus subtilis* (2OQC). Ntn-hydrolases with reported bile salt hydrolase activity: *Bl*BSH, from *Bifidobacterium longum* (2HF0), and *Ef*BSH, from *Enterococcus feacalis* (4WL3). Ntn-hydrolases with reported cyclic lipopeptide acylase activity were as follows: *Ss*CLA, from *Streptomyces* sp. FERM-BP5809 (BAD07025). Bifunctional acylases (with both penicillin and AHL acylase activities) are indicated by asterisks and, among them, *Sl*PVA and *Au*AAC present aculeacin A acylase activity as well.

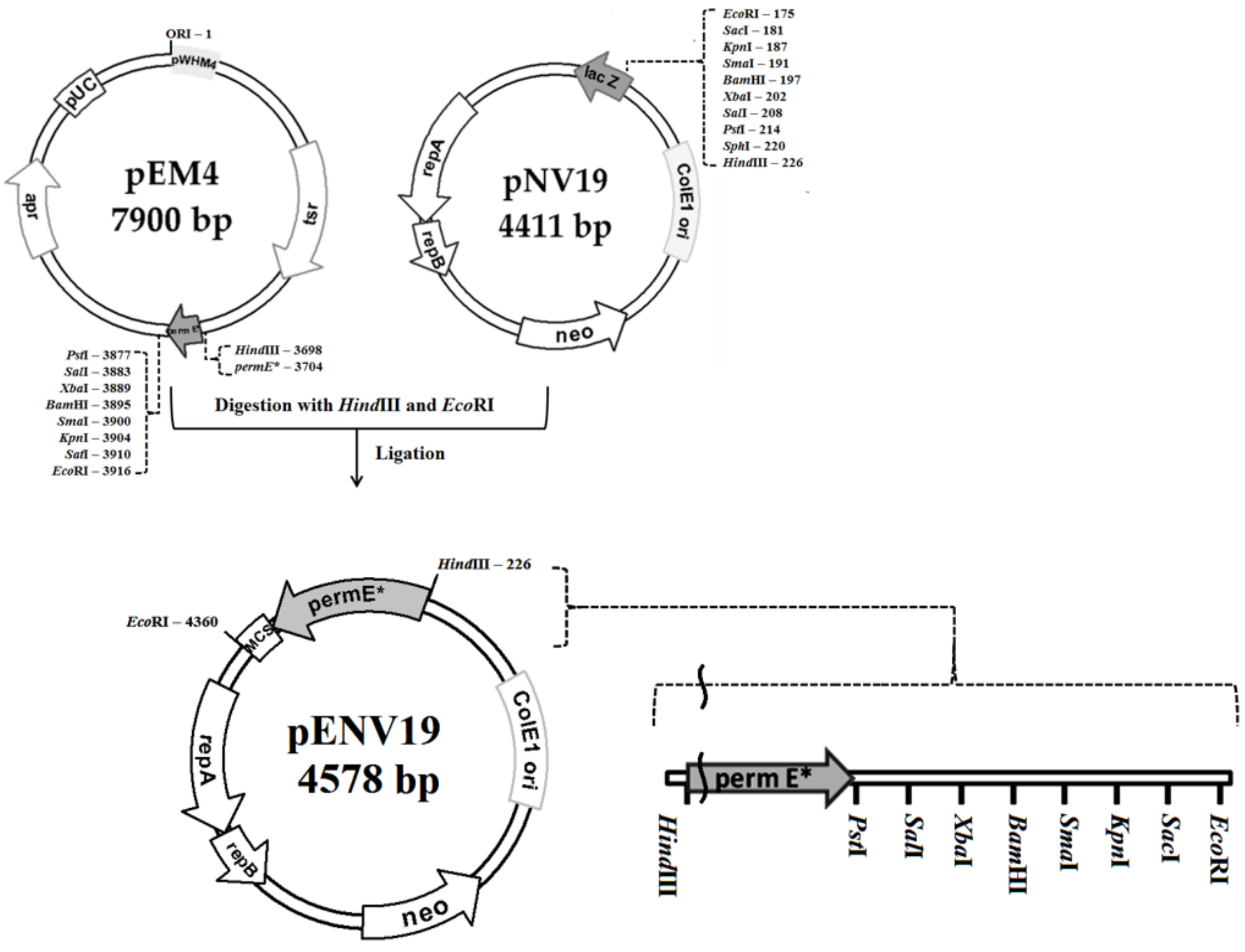


gaattcgagctcggtaccccgggggatcctctagagtcgacctgcagtaccaaccggcacgattgtgcccacaacagcatcgcggtgcc

EcoRI SstI KpnI SmaI BamHI XbaI SalI PstI

acgtgtggaccgcgtcggtcagatcctccccgcacctctcgccagccgtcaagatcgaccgcgtgcacctgcgatcgccgatcaaccgcg

actagcatcgggcgcaagccgccactcgaacggacactcgcaagctt

HindIII

**Supplementary Figure S4.** Shuttle vector pENV19 construction.
